# Alcohol pretreatment of stools effect on culturomics

**DOI:** 10.1038/s41598-020-62068-x

**Published:** 2020-03-23

**Authors:** Pamela Afouda, Marie Hocquart, Thi-Phuong-Thao Pham, Edmond Kuete, Issa Isaac Ngom, Niokhor Dione, Camille Valles, Sara Bellali, Jean-Christophe Lagier, Grégory Dubourg, Didier Raoult

**Affiliations:** 1 Aix Marseille Université, IRD, AP-HM, MEPHI, Marseille, France; 2 IHU Méditerranée Infection, Marseille, France

**Keywords:** Bacteriology, Bacteriology, Bacteriology, Microbiome, Microbiome

## Abstract

Recent studies have used ethanol stool disinfection as a mean of promoting valuable species’ cultivation in bacteriotherapy trials for *Clostridium difficile* infections (CDI) treatment with a particular focus on sporulating bacteria. Moreover, the culturomic approach has considerably enriched the repertoire of cultivable organisms in the human gut in recent years. This study aimed to apply this culturomic approach on fecal donor samples treated with ethanol disinfection to evidence potential beneficial microbes that could be used in bacteriotherapy trials for the treatment of CDI. Thereby, a total of 254 bacterial species were identified, 9 of which were novel. Of these, 242 have never been included in clinical trials for the treatment of CDIs, representing potential new candidates for bacteriotherapy trials. While non-sporulating species were nevertheless more affected by the ethanol pretreatment than sporulating species, the ethanol disinfection technique did not specifically select bacteria able to sporulate, as suggested by previous studies. Furthermore, some bacteria previously considered as potential candidates for bacteriotherapy have been lost after ethanol treatment. This study, while enriching the bacterial repertoire of the human intestine, would nevertheless require determining the exact contribution of each of species composing the bacterial consortia intended to be administered for CDI treatment.

## Introduction

*Clostridium difficile* infection (CDI) represents a public health problem worldwide as it is associated with significant morbidity and mortality^1–4^. This infection, due to the establishment of toxigenic *C. difficile* in the human digestive tract is a consequence of intestinal microbiota imbalance^5^ due to antibiotic intake. Until recently, the administration of antimicrobial agents was the treatment of choice for this type of infection and therefore exposed patients to the risk of recurrence of CDI^6^. The modification of the gut microbiota during antibiotic treatment induces an increase in the production of succinate. Indeed, *Clostridium difficile* can use this succinate produced by converting it into butyrate, thus promoting its colonization of the host’s intestine^7^. Otherwise, alternative treatments to antibiotics are increasingly being used, such as the use of monoclonal antibodies against toxins produced by *Clostridium difficile*, vaccination against *Clostridium difficile* infection, transplantation of non-toxic strains of *Clostridium difficile*, but also the use of transplants of healthy faecal microbiota from healthy subjects^8–10^. Fecal microbiota transplantation for the treatment of CDI recurrence has been shown to be effective in recent years^11–14^. However, its non-standardization and its unattractive character^15^ have led to the emergence of studies on bacteriotherapy, which consists of using non-toxic, bacterial cocktails, sporulating or not, isolated from the feces of fecal transplant donors to treat or prevent CDI recurrence^9,16–18^. Several mixtures of bacterial strains (previously known or new species) have already been proposed, mainly species belonging to phyla *Firmicutes* and *Bacteroidetes*, with an interesting efficacy in the majority of patients treated^16–20^. Otherwise, the treatment of clinical samples or mixed cultures with ethanol has been described as very effective for the isolation of sporulated bacteria^18,21^. A recent study used disinfection of donor stools with ethanol^21^ before bacterial selection to eliminate vegetative forms, resulting in the identification by metagenomics of very few organisms (i.e., 34 genera of bacteria)^18^. However, metagenomics does not allow distinguishing alive from dead bacteria, nor does it provide biological material for the bacteriotherapy approach. As part of the culturomic approach that has substantially increased the bacterial diversity associated with human in recent years^22,23^, we propose herein to apply this technique to fecal stool transplant donors. This would allow to assess (i) which microbes are remaining after such disinfection and (ii) to obtain biological material for those that could be included as part of a bacteriotherapy strategy.

This work therefore consists of an exhaustive analysis of the fresh intestinal microbiota of 8 fecal transplant donors and 3 samples of stool infusions from fecal transplant donors after pretreatment with ethanol using the culturomic approach. The objective is to evaluate the panel of bacterial species isolated from these stool samples that have not been reported in previous studies and would have a potential therapeutic effect on *Clostridium difficile* infections (CDI).

## Results

### Distribution of bacterial species

**Figure 1:**
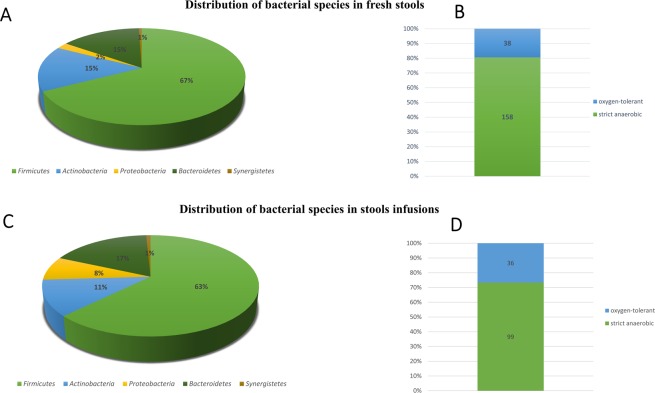
(**A**) Classification in phylum of the 196 bacteria species of the 8 fresh stools of fecal transplant donors pretreated with ethanol. (**B**) Oxygen tolerance of the 196 bacteria species of the 8 fresh stools of fecal transplant donors pretreated with ethanol. (**C**) Classification in phylum of the 135 bacteria species of the 3 fecal infusions of fecal transplant donors pretreated with ethanol. (**D**) Oxygen tolerance of the 135 bacteria species of the 3 fecal infusions of fecal transplant donors pretreated with ethanol.

The 16 enrichment conditions (Supplementary Table 1) and 6 different directs cultures used allowed us to test a total of 38,016 bacterial colonies by MALDI-TOF MS among the 8 fresh stool specimens. As a result, 196 bacterial species were identified, of which 99 were known in the human gut (50%), 13 in humans but not in the gut (7%), 12 unknown from human being (6%), while 63 were new species previously discovered as part of other culturomics studies (32%)^23^ and 9 were new species discovered in this study (5%) (Appendix 1). The classification in phylum shows a predominance of *Firmicutes* (67%), followed by *Actinobacteria* (15%), *Bacteroidetes* (15%) and small portions of *Proteobacteria* (2%) and *Synergistetes* (1%) (Fig. 1A). These species are mostly anaerobes (158/196 = 80.61%) (Fig. 1B). Concerning the 3 fecal infusions, a total of 16,500 colonies were tested by MALDI-TOF MS, representing 135 different species, 81 of which are already known in the human gut (60%), 12 in the human, but not in gut (9%), 8 were non-Human (6%), 34 were culturomics new species (25%), but no other new species was discovered. The same profile of phylum distribution is observed in the fecal infusions. *Firmicutes* represent 63%, *Bacteroidetes* 17%, *Actinobacteria* 11% followed by small portions of *Proteobacteria* (8%) and *Synergistetes* (1%) (Fig. 1C). The majority were also anaerobes (73.33%) (Fig. 1D). A total of 254 bacterial species were isolated from 11 stools samples disinfected with ethanol, mainly anaerobes (194/254 = 73.83%) (Appendix 1). These species are predominated by the families *Clostridiaceae* (37/254 = 14.57%), *Ruminococcaceae* (17/254 = 6.69%), *Bacteroidaceae* (16/254 = 6.30%), *Bacillaceae* (15/254 = 5.91%) and *Lachnospiraceae* (13/254 = 5.12%) (Appendix 1). The richness of bacterial species obtained in stools is very variable from one stool to another (from 35 to 68 species for fresh stool and 33 to 83 for fecal infusions) (Appendix 2).

Overall, for each fresh stool, the proportion of new species previously known in culture and those added in this study represents a little more than one third (72/196 species = 36.73%) of the total proportion of bacterial species obtained (Appendix 1), and those found in fecal infusions represent 1/4 (34/135 species; 25.18%) of these total species (Appendix 1). For the 11 samples combined, the proportion of new species previously isolated in culturomics and those discovered following ethanol disinfection, represent 32.67% (83/254) of the total proportion of bacterial species (Appendix 1).

### Impact of ethanol disinfection

**Figure 2:**
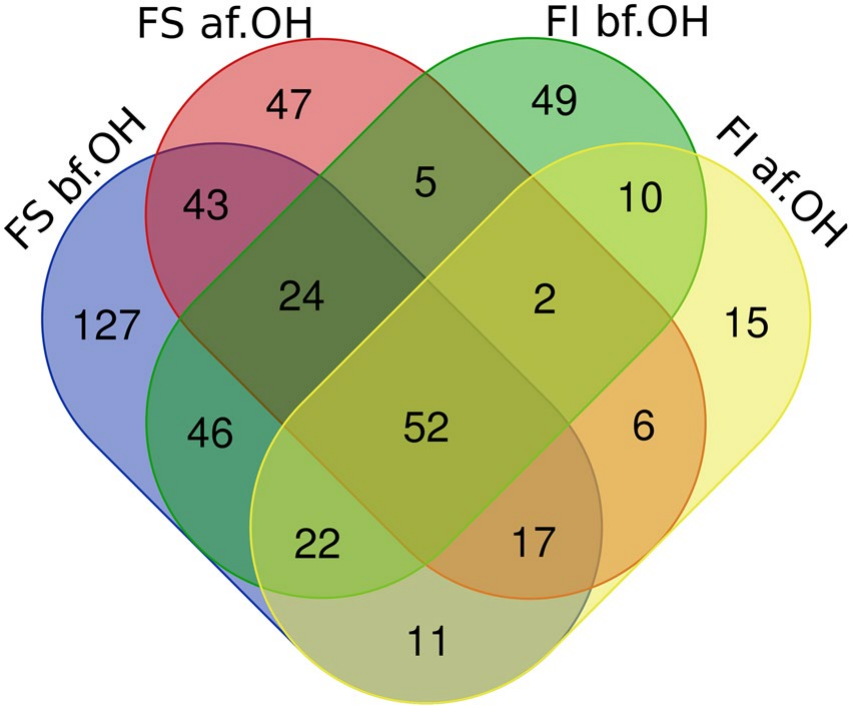
Venn diagram of the bacterial species obtained before and after ethanol disinfection: FS bf. OH = bacterial species obtained in the 8 fresh stools before ethanol disinfection; FI bf. OH = bacterial species obtained in the 3 fecal infusions before ethanol disinfection; FS af. OH = bacterial species obtained in the 8 fresh stools after ethanol disinfection; FI af. OH = bacterial species obtained in the 3 fecal infusions after ethanol disinfection.

**Figure 3:**
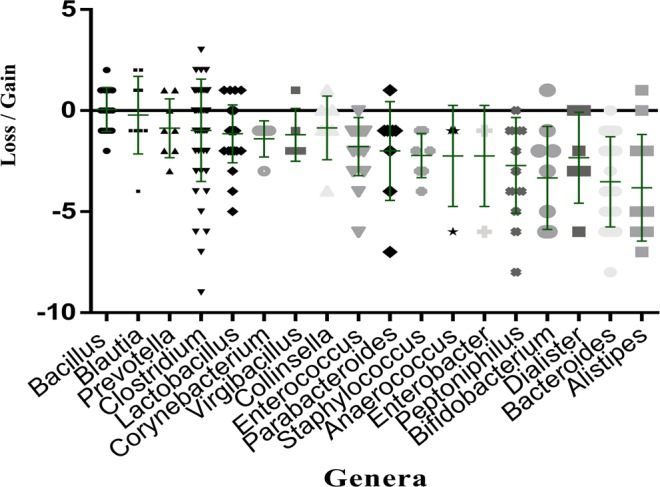
Graphical representation of the mean impact of ethanol disinfection toward several bacterial genera. The mean impact was assessed by summing the number of samples for which the genus was gained and subtracting the number of samples for which it was lost. Each loss corresponds to the sum of the number of species belonging to this genus present before disinfection, but absent after disinfection. Each gain corresponds to the sum of the number of species belonging to this genus isolated absent before disinfection, but present after disinfection.

The 18 usual culturomics conditions were carried out in parallel with those following ethanol disinfection on the same fecal samples. To assess the impact of ethanol disinfection, we compared the culture data obtained before and after ethanol disinfection of the same stool sample. Ethanol disinfection applied to the 8 fresh samples allows 60 species that were absent under the 18 standard cultivation conditions to be cultivated, the same figure being 49 species for the 3 fecal infusions (Fig. 2, Appendix 1). Considering all bacterial species isolated in the 11 samples, 68 bacterial species were unique to ethanol disinfection, while 98 and 329 different species are acquired and lost at least once, respectively (Appendix 2). In detail, ethanol disinfection has eliminated bacteria such as *Phascolarctobacterium faecium* (Supplementary Table 2) and *Barnesiella intestinihominis* (Appendix 2), but also several species of the genera *Alistipes*, *Bacteroides*, *Dialister*, *Bifidobacterium* for which the mean differential frequency of the different species are respectively −3.81, −3.52, −3.50 and −3.33 (Fig. 3, Table 1). Species belonging to the genera *Bacillus*, *Clostridium*, *Blautia*, *Lactobacillus* and *Prevotella* seem to be less affected by this bacterial elimination caused by ethanol disinfection (Fig. 3, Table 1). All species gained and lost in each stool with ethanol disinfection are illustrated in Appendix 2. More particularly, at the family level, we observed after ethanol disinfection an enrichment in *Ruminococcaceae*, *Bacteroidaceae* and *Lachnospiraceae*, whose rates decreased respectively from 2.70%, 5.16% and 4.18% before disinfection to 6.69%, 6.30% and 5.12% after disinfection (Appendix 1).

**Table 1:** Bacterial genera for which at least 4 different bacterial species are lost under the ethanol conditions.

Genera	Number of species lost	Number of total species	Average of total species	Standard deviation
*Bacillus*	5	19	0.055	1.05
*Blautia*	4	9	−0.22	1.92
*Prevotella*	5	8	−0.87	1,45
*Clostridium*	21	47	−0.97	2,53
*Lactobacillus*	20	27	−1.14	1.43
*Corynebacterium*	5	5	−1.40	0.89
*Virgibacillus*	4	5	−1.75	0.50
*Collinsella*	4	7	−0.85	1.57
*Enterococcus*	17	18	−1.77	1.43
*Parabacteroides*	7	8	−2.00	2.44
*Staphylococcus*	9	9	−2.22	1.09
*Anaerococcus*	4	4	−2.25	2.50
*Enterobacter*	4	4	−2.25	2.50
*Peptoniphilus*	13	14	−2.71	2.36
*Bifidobacterium*	8	9	−3.33	2.54
*Dialister*	4	6	−2.33	2.25
*Bacteroides*	20	21	−3.52	2.22
*Alistipes*	9	11	−3.81	2.63

### Impact on spore forming species

**Figure 4:**
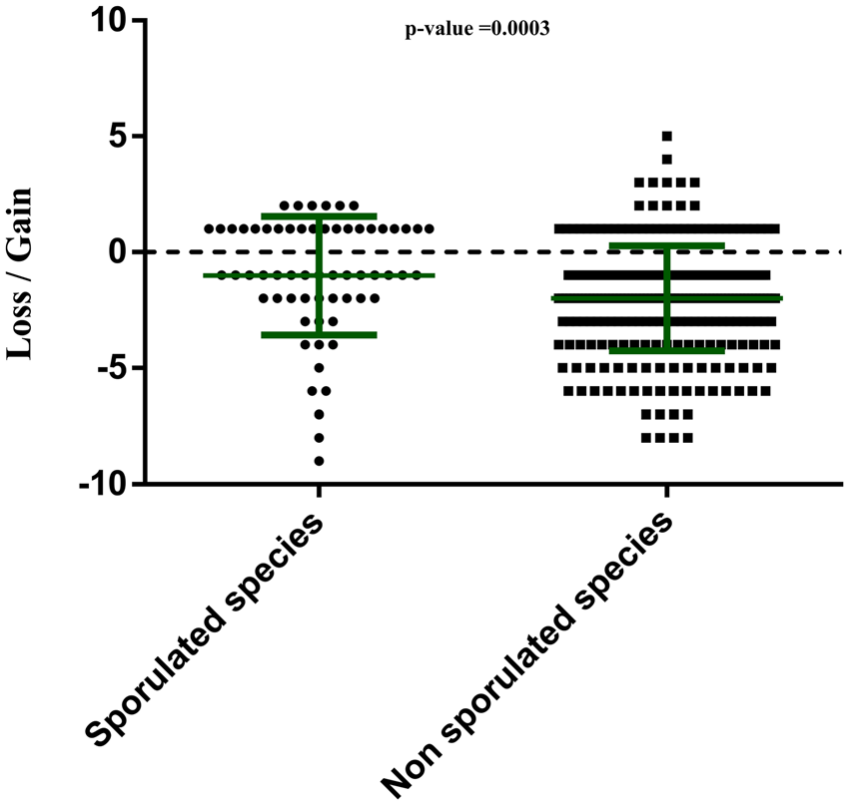
Graphical representation of the mean impact of ethanol disinfection on sporulated and non-sporulated species. Each point represents a species that has been classified as sporulated or non-sporulated. We have assessed the mean impact of ethanol disinfection for each species by summing the number of samples for which the species was gained and subtracting the number of samples for which it was lost. A gain corresponds to a species absent before disinfection, but recovered after disinfection, while a loss corresponds to a species present before disinfection, but absent after disinfection. Error bars are shown in green; p-value = 0.0003 with Mann-Whitney test.

When all samples are combined, the number of species gained at least once with ethanol disinfection was 98, 26 of which (26.53%) were sporulating species. Among the 329 species lost at least once with ethanol disinfection, 10.94% (36 species) were sporulating species (Appendix 2, Fig. 4). In addition, only 32.65% of the species found in both ethanol and ethanol-free conditions are sporulating (16 species). While these data suggest that ethanol incubation does not select only spore-forming species, non-spore-forming species are nevertheless more affected by ethanol pre-treatment than spore-forming species(p = 0.0003) (Fig. 4).

### Impact of the culturomics strategy on the cultivation of potential bacterial species of interest for bacteriotherapy

**Figure 5:**
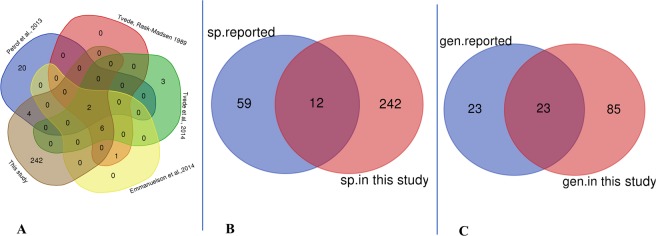
Venn Diagram comparing bacterial strains known previously in bacteriotherapy with those obtained in this study; (**A**) Separate comparison of all bacterial species previously known against those of this study; two bacterial species are shared between all studies. These are: *Escherichia coli* and *Bacteroides ovatus*. (**B**) Grouped comparison of all bacterial species previously known in bacteriotherapy against those of this study; 12 bacterial species are shared between these two groups; these are: *Clostridium ramosum*, *Enterococcus fecalis*, *Clostridium bifermentans*, *Escherichia coli*, *Collinsella aerofaciens*, *Clostridium innocuum*, *Bifidobacterium longum*, *Bacteroides thetaiotaomicron*, *Bacteroides vulgatus*, *Bacteroides ovatus*, *Parabacteroides distasonis* and *Bifidobacterium adolescentis*. (**C**) Grouped comparison of all previously known bacterial genera in bacteriotherapy against those of this study; 23 bacterial genera are shared between these two groups. These are genera *Bifidobacterium*, *Streptococcus*, *Dorea*, *Terrisporobacter*, *Turicibacter*, *Ruminiclostridium*, *Enterococcus*, *Bacteroides*, *Parabacteroides*, *Anaerostipes*, *Hungatella*, *Clostridium*, *Anaerotruncus*, *Anaerofustis*, *Flavonifractor*, *Ruminococcus, Escherichia*, *Intestinibacter*, *Oscillibacter*, *Eubacterium*, *Collinsella*, *Lactobacillus* and *Blautia*.

Of the 71 species previously reported in bacteriotherapy trials^16–20^, 12 (17%) were recovered in this study (Fig. 5A,B). The species are as follows: *Bacteroides ovatus*, *Bacteroides thetaiotaomicron*, *Bacteroides vulgatus*, *Bifidobacterium adolescentis*, *Bifidobacterium longum*, *Clostridium bifermentans*, *Clostridium innocuum*, *Clostridium ramosum*, *Collinsella aerofaciens*, *Enterococcus fecalis*, *Escherichia coli* and *Parabacteroides distasonis*. In addition, 242 bacterial species isolated in this study were not considered in bacteriotherapy trials (Fig. 5B, Appendix 3). These are mainly strict anaerobes (185/242 = 76.45%) and predominated by phyla *Firmicutes* (158/242), *Bacteroidetes* (33/242) and *Actinobacteria* (31/242) and with a low proportion of *Proteobacteria* and *Synergistetes* (Appendix 3). Of the 59 bacterial species absent from this study, 6 were actually collected from samples prior to ethanol disinfection (i.e., as part of the 18 standard culture conditions), suggesting that they did not survive the disinfection procedure (i.e., *LactobacilIus rhamnosus*, *Faecalibacterium prausnitzii*, *Acidaminococcus intestinalis*, *Dorea longicatena*, *Streptococcus mitis* and *Lactobacillus paracasei*) (Appendix 1, Appendix 3).

### Succinate production

**Figure 6:**
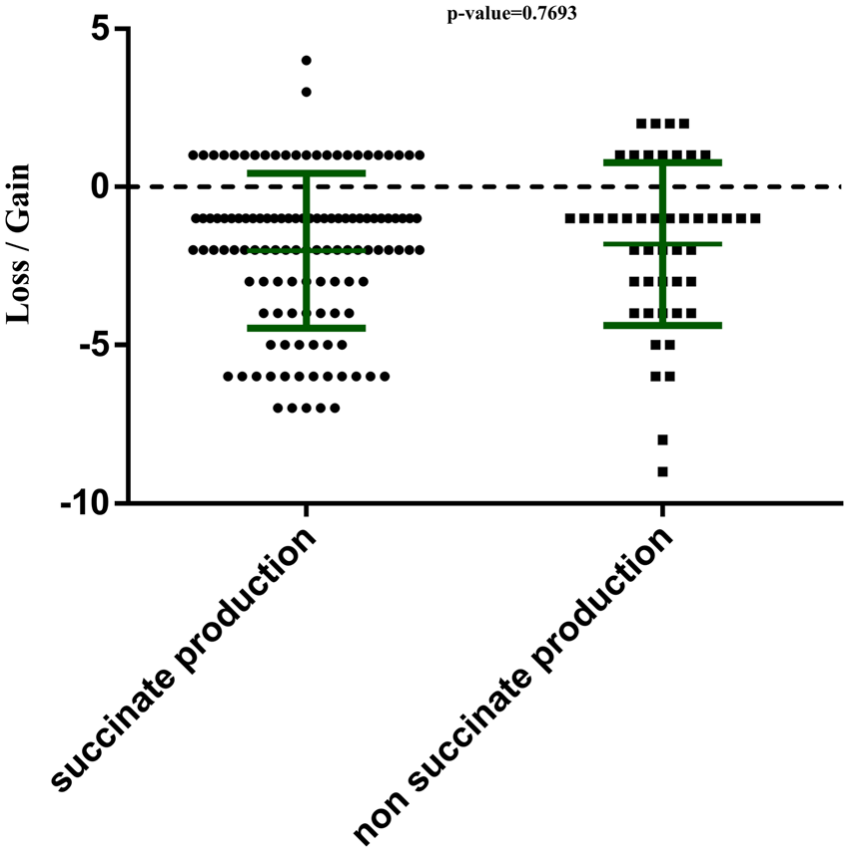
Graphical representation of the mean impact of ethanol disinfection on succinate producers and non-succinate producers. We have assessed the mean impact of ethanol disinfection for each species by summing the number of samples for which the species was gained and by subtracting the number of samples for which it was lost. A gain corresponds to a species absent before disinfection, but recovered after disinfection, while a loss corresponds to a species present before disinfection but absent after disinfection. Error bars are shown in green; p-value = 0.7693 with Mann-Whitney test. Error bars are shown in green; p-value = 0.7693 with Mann-Whitney test.

As high levels of succinate within the gut microbiota could promote CDI^7^, we assessed the ability from bacteria isolated as part of this study to produce succinate. Considering the species cultured following ethanol disinfection or not, we obtained information for 158/427 species. Of these, 112 were succinate-producing bacteria and 46 were non-succinate-producing bacteria (Appendix 2). However, no significant difference was observed in the impact of ethanol disinfection on succinate-producing species compared to species unable to produce succinate (Mann-Whitney test; p = 0.7693) (Fig. 6).

### New species

The new genera and species found in this study were all found for the first time in fresh stools and were named as follows: *Massiliimalia timonensis* (=CSUR P3753 = CCUG 7163), *Lactobacillus timonensis* (=CSUR P3825 = CCUG 70711), *Ethanolibacter massiliensis* (=CSUR P5640 = CECT 9563), *Prevotella merdae* (=CSURP4119 = CECT9566), *Ruminococcus merdae* (=CSUR P4123), *Clostridium cacamassiliense* (=CSUR P5205), *Dialister massiliensis* (=CSURP5638), *Neochristensenella massiliensis* (=CSURP4260) and *Pseudoruminocossus massiliensis* (=CSURP3876). Of these, only *Massiliimalia timonensis* was found in half of the fresh stool samples. The others were found either in 3 (*Ruminococcus merdae*), 2 (*Lactobacillus timonensis*), or in one stool sample at a time (*Ethanolibacter massiliensis*, *Prevotella merdae*, *Clostridium cacamassiliense*, *Dialister massiliensis*, *Neochristensenella massiliensis*, *Pseudoruminococcus massiliensis*) (Appendix 2). None of these species were found in the 3 fecal infusions.

## Discussion

Herein, we carried out a complete culture analysis of 11 fecal samples after ethanol treatment using a culturomics approach that will then be used as bacteriotherapy, particularly for patients with *Clostridium difficile* infections (CDI). As a result, we cultured a total of 254 species mostly anaerobic, of which 68 bacterial species (containing 9 new bacterial taxa) were obtained only after treatment with ethanol (Appendix 1).

As disinfection technique has already been reported in a bacteriotherapy trial for the treatment of *Clostridium difficile* infections^18^, in particular for the selection of sporulated bacteria^21^, the primary aim of this study was to identify potential bacterial species that could be used for bacteriotherapy trials through the culturomics approach^22,23^. Secondly, this work could be used to assess the potential relevance of this culture condition to cultural studies.

Focusing on the contribution of the culturomics strategy to the recapture of bacteria of interest in bacteriotherapy, we found that, compared to previous studies, 23 bacterial genera are shared between the two groups, with 85 bacterial genera representing at least 242 species being reported only in this study (Fig. 5A–C, Appendix 3). This large difference can be explained on the one hand, by the size of our sampling, which is much more important than in the previous studies, and, on the other hand, by our culturomics strategy, which targets the cultivation of a large number of fastidious species. On the other hand, the genera *Coprobacillus*, *Lachnoclostridium*, *Acidaminococcus*, *Peptostreptococcus*, *Faecalibacterium*, *Tyzzerella*, *Coprococcus* and *Holdemanella*, previously identified as candidates^16–20^ and found under culturomics conditions prior ethanol disinfection, were all eliminated after ethanol disinfection protocol. Similarly, the *LactobacilIus rhamnosus*, *Faecalibacterium prausnitzii*, *Acidaminococcus intestinalis*, *Dorea longicatena*, *Streptococcus mitis* and *Lactobacillus paracasei* species do not appear to have survived this disinfection protocol. These findings could appear counterintuitive as *Lactobacillus* species are frequently included in probiotic formulations for preventing CDI relapses, while *Faecalibacterium prausnitzii* has been used in a bacteriotherapy trial aiming to eradicate CDI in two patients^16–20^. As these microbes are often administered in combination^24^, the exact contribution of each of its species to the treatment or prevention of CDI remains undetermined and requires further studies.

When focusing on bacterial taxa dramatically affected by ethanol disinfection, species such as *Phascolarctobacterium faecium* and *Barnesiella intestinihominis* (Appendix 2), but also some species of the genera *Alistipes*, *Bacteroide*s and *Bifidobacterium* were strongly eliminated (Table 1, Fig. 3). Apart from the *Bacteroides* and *Bifidobacterium* genera previously reported in bacteriotherapy for the treatment of *Clostridium difficile* infections, the others have not been found in any bacteriotherapy studies^16–20^, although it has been reported that many of these anaerobes are described as essential for human intestinal homeostasis^25–29^. These results may suggest that the majority of these ethanol-eliminated bacterial species would not be fully essential for the treatment of *Clostridium difficile* infections. Among the genera least affected by ethanol disinfection in this study, *Blautia, Clostridium* and *Lactobacillus* were identified as bacteriotherapy candidates for the treatment of *Clostridium difficile* infections^16–20^. Indeed, ethanol stool disinfection would therefore select the majority of bacterial genera sufficient to restore the diversity of the gut microbiota in the treatment of *Clostridium difficile* infections.

Interestingly, ethanol disinfection has enriched the proportion of species belonging to the *Ruminococcaceae* and *Lachnospiraceae* (Appendix 1, Appendix 4). These two bacterial families have been suggested to be predictive of favorable outcome following FMT for treating *Clostridium difficile* infections^30^. Our list of bacteria obtained with ethanol disinfection is therefore quite consistent and contains probable candidates for CDI bacteriotherapy trials.

Strikingly, sporulated bacterial species only represent 26.53% of all the species gained by ethanol disinfection (Appendix 2), and the majority of species gained at least 3 times are new non-spore forming culturomics species^23^ (Supplementary Table 3). This highlights the fact that ethanol disinfection is ultimately an effective approach to recover fastidious and minority species that have not been found under standard culturomics conditions^22,23^ for the same samples and is therefore suitable for the exploration of the human gut microbiota. However, our data do not support the idea that only sporulating species can survive this procedure (Fig. 4), as previously suggested^18,21^, even though they have been less affected by ethanol disinfection than non-sporulating species.

Furthermore, some anaerobic bacteria reported in this study produce^31–34^ or consume^28,35^ succinate (Appendix 2). This production of succinate by intestinal bacteria modulates glucose metabolism in the healthy host by inducing activation of intestinal gluconeogenesis^36^. However, in the host suffering from CDI, *Clostridium difficile* can exploit the succinate produced by converting it into butyrate to multiply and exert an increased pathogenic effect^7^. In our study, ethanol disinfection has no particular effect on the succinate producing species as they were eliminated by ethanol incubation as much as non-succinate producing species (Fig. 6).

Finally, considering the gains and losses of stool bacterial species in each stool after ethanol treatment, we noted that 98 minority bacterial species are gained at least once, versus 329 majority species lost at least once (Appendix 2). This suggests a complementarity between standard culturomics conditions and ethanol disinfection conditions for the isolation of high numbers of microorganisms from stool samples. Indeed, ethanol stool disinfection may therefore be an additional condition to be added to our laboratory culture strategy for future studies, to explore and increase the microbial flora not yet cultivated in order to enrich the bacterial repertoire of rare bacterial species. While the alcohol disinfection was empirically designed to select the sporulated bacteria, another point of consideration could be to explore the impact of heat shock on fecal specimen from donors to search for new bacteriotherapy trials candidates.

## Conclusion

In conclusion, we demonstrated here that ethanol disinfection associated with the culturomic approach could be a promising approach to explore the diversity of the human gut microbiota by selecting bacterial species of interest, which can be potentially usable in bacteriotherapy. High-scale culture approach applied to 11 samples allowed us to isolate 242 species that have never been reported in previous bacteriotherapy trials and that could be the subject of further studies in the treatment of *Clostridium difficile* infections.

## Material and Methods

### Samples information

The material consists of 11 samples of healthy subjects, 8 of which represent fresh stools from fecal transplant donors and 3 were samples of fecal infusion obtained from frozen stools (80 °C). These 11 samples were collected from 9 different fecal donors: the 8 fresh stools represent 8 different donors, fecal infusions 1 and 2 were collected from donors of fresh stools 1 and 4, and fecal infusion 3 was obtained from the ninth donor (Supplementary Table 4). Each fecal donor gave informed and signed consent. The study was approved by the ethics committee of the Institut Hospitalo-Universitaire-Méditerranée Infection under agreement number 2016–011 and all the methods were performed in accordance with relevant and regulations.

### Standard microbiological procedures

According to the French Recommendations of the National Agency for the Safety of Medicines (ANSM)^37^, the stool have been qualified before being used for fecal transplantation. This qualification procedure includes the search for pathogens and for transferable resistance mechanisms from the stool and blood of donor. Any positive result to a pathogen or resistance mechanisms precludes the use of this donor’s stool (Supplementary Table 5).

### Preparation of fecal infusion

Fecal infusion is a stool donation prepared to be transplanted to a receiver, while fecal transplant is the action of transplanting the fecal infusion to a receiver. Between September 2016 and December 2017, 3 different fecal infusions were prepared according to the procedure previously reported^38^ and from 3 different frozen fecal transplant donor stools. Donors were, pre-selected from questionnaires and medical tests according to the ANSM^37^ (Supplementary Data). Briefly, for the preparation of each fecal infusion, each donor’s stool is thawed at room temperature for 4 hours. In a pitcher, 500 mL of saline is added to the stool. The mixture is then mixed for 5 minutes and passed through a sieve having a pore size of 1 mm in diameter. The fecal infusion is collected in 10 mL syringes and then kept under anaerobic conditions (ie in a plastic bag + GENbag Anaer systems (bioMérieux)). Each fecal infusion represents one sample and one donor.

### Process

Manipulations of the 11 samples used (8 fresh stools samples and 3 fecal infusions samples) were performed under microbiological hood and anaerobic chamber. Each stool was disinfected separately with ethanol according to a previous study^18^ in order to eliminate vegetative forms as much as possible to promote the growth of bacteria resistant to alcohol^18^ and capable of sporulating^18,21^. To this end, about 10 g of each stool was homogenized in 10 mL of saline solution (NaCL 0.9%, Versylene® Fresenius, Sevres, France). Then, the mixture is filtered through a sieve with a pore size of 1 mm in diameter and the supernatant is recovered. In falcon tubes, 10 mL of 100% ethanol is added to 10 mL of supernatant containing the bacterial cells and the spores. This mixture is then incubated at room temperature under anaerobic conditions for 1 hour. Thereafter, the mixture is centrifuged for 2 minutes at 5000 rpm to remove ethanol (which in this case is the supernatant). The pellet was washed twice with saline solution by centrifugation to remove any trace of the remaining ethanol before proceeding to microbial culturomics.

### Microbial culturomics

After ethanol disinfection of the 11 samples, we performed, the culture on 6 different solid culture media and, on 16 different pre-enrichment conditions (Supplementary Table 1), which will then be subcultured on sheep blood-enriched Columbia agar (COS) medium (bioMérieux, Marcy l’Etoile, France). A total of 22 culture conditions were thereby used in this study. Briefly, the direct culture after ethanol treatment was carried out in anaerobic chamber on these 6 different types of culture media: Reinforced clostridial agar (HiMedia™ Laboratories Pvt Lt, India), Wilkins Chalgren agar (Becton, Dickinson company, Le Pont-de-Claix, France), Brain-heart infusion agar (Becton, Dickinson company, Le Pont-de-Claix, France), deMan, Rogosa and Sharpe agar (Sigma-Aldrich, Saint-Louis, USA)^16^, 5% sheep blood-enriched columbia agar (COS) (bioMérieux, Marcy l’Etoile, France), Yeast Extract-Casein Hydrolysate-Fatty Acids (YCFA) agar, according to the composition previously described^39^, supplemented with 0.002 g/ml each of glucose, maltose, cellobiose and 0.1% sodium taurocholate^40^. In parallel, the stools were pre-incubated in blood culture bottles supplemented or not with 5% of the rumen, 5% of blood or both under aerobic and anaerobic conditions at 37 °C and then at 28 °C, under 16 selected culturomics conditions^22,23^. These blood cultures were seeded every 3 days on Columbia agar with 5% sheep blood (bioMérieux, Marcy l’Etoile, France) under aerobic and anaerobic conditions for 30 days^22,23^ (Supplementary Table 1). All the morphologically different colonies obtained in direct culture and preincubation were subcultured on COS and bacterial identification was performed after 24–72 hours of incubation. The subcultures were identified using MALDI-TOF mass spectrometry with a Microflex LT spectrometer (Bruker Daltonics, Leipzig, Germany) as previously described^41^. When identification was not possible by MALDI-TOF, 16S rRNA gene sequencing was performed on unidentified colonies.

### 16S rRNA gene sequencing

DNA extraction was performed using the EZ1 DNA Tissue Kit and BioRobot EZ1 Advanced XL (Qiagen, Courtaboeuf, France). DNA extracts were used for 16S rRNA amplification using the fD1 and rP2 primers (Eurogentec, Angers, France). Amplicon sequencing was performed using the Big Dye® Terminator v1.1 Cycle Sequencing Kit and an ABI Prism 3130xl Genetic Analyzer capillary sequencer (Applied Biosystems), as previously described^42^. The obtained 16S rRNA sequences were compared with those available in GenBank (http://www.ncbi.nlm.nih.gov/genbank). Identification at the species level was defined by a 16S rRNA gene sequence similarity ≥98.65% with the sequence of the prototype strain of a species with standing in nomenclature. When this percentage of identity was lower than the generally accepted thresholds of 98.65% or 95%, the strain studied was considered a putative new species or genus, respectively^43,44^.

### Succinate production and sporulation

The “Google” search engine was used to search for data on the production or non-production of succinate and sporulation of our isolated bacterial species in this study using the keywords “name of bacteria” followed by “succinate production” or “spore”. For species for which full descriptions or work on succinate and spore production were available, searches were carried out using “PubMed” and “Google scholar” databases, but also using “List of Prokaryotic names with Standing in Nomenclature” (http://www.bacterio.net/). Concerning the different new species of “culturomics” found in this study, we used our laboratory data (published or not).

### Venn diagrams

Venn diagrams comparing the bacterial species obtained in this study with those previously reported in bacteriotherapy were produced online at: http://bioinformatics.psb.ugent.be/webtools/Venn.

### Statistical test

Plots and statistical analyses were performed using the GraphPad Prism software (version 6.01; GraphPad Software, Inc., www.graphpad.com) for Figs. 3, 4 and 6 based on the number of bacterial taxa gained and lost in the 11 stools samples (Appendix 2).

## Supplementary information

Supplementary data.

Appendix 1.

Appendix 2.

Appendix 3.

Appendix 4.

## Data Availability

Additional data on the bacterial species isolated in this study are presented in Appendix 1 to 4. Supplementary Tables and Appendices legends are available in “Supplementary Data”.
