# Supplementary material for: Alcohol pretreatment of stools effect on culturomics
Source: Sci Rep. 2020 Mar 23;10:5190. doi: 10.1038/s41598-020-62068-x (PMC7089995; doi:10.1038/s41598-020-62068-x)
Supplement: Supplementary file 1 — Supplementary data. [file 41598_2020_62068_MOESM1_ESM.pdf]

Supplementary data

Alcohol pretreatment of stools effect on culturomics

Pamela AFOUDA[1,2], Marie HOCQUART[1,2], Thi-Phuong-Thao PHAM[1,2], Edmond KUETE[1,2], Issa Isaac NGOM[1-2], Niokhor DIONE[1-2], Camille VALLES[1-2], Sara BELLALI[1,2], Jean-Christophe LAGIER[1,2], Grégory DUBOURG[1,2] and Didier RAOULT[1,2]*

[1] Aix Marseille Université, IRD, AP-HM, MEPHI, Marseille, France

[2] IHU Méditerranée Infection, Marseille, France

*Corresponding author : Prof. Didier RAOULT

Adresse : IHU, 19-21 boulevard Jean Moulin, 13005, Marseille, France

Tel : +33 413 732 401

Fax: +33 413 732 402

Supplementary Table 1. Enrichment conditions used

16 culture conditions
Preincubation in aerobic blood culture bottle with 5ml rumen fluid and 5ml sheep blood, Aerobic condition, 37°C
Preincubation in anaerobic blood culture bottle with 5ml rumen fluid and 5ml sheep blood, Anaerobic condition, 37°C
Preincubation in aerobic blood culture bottle with 5ml rumen fluid, Aerobic condition, 37°C
Preincubation in anaerobic blood culture bottle with 5ml rumen fluid, Anaerobic condition, 37°C
Preincubation in aerobic blood culture bottle with 5ml sheep blood, Aerobic condition, 37°C
Preincubation in anaerobic blood culture bottle with 5ml sheep blood, Anaerobic condition, 37°C
Preincubation in aerobic blood culture bottle with 5ml rumen fluid and 5ml sheep blood, Aerobic condition, 28°C
Preincubation in anaerobic blood culture bottle with 5ml rumen fluid and 5ml sheep blood, Anaerobic condition, 28°C
Preincubation in aerobic blood culture bottle with 5ml sheep blood, Aerobic condition, 28°C
Preincubation in anaerobic blood culture bottle with 5ml sheep blood, Anaerobic condition, 28°C
Preincubation in aerobic condition in trypticase soy broth, with 5% sheep blood, Aerobic condition, 37°C
Preincubation in anaerobic condition in trypticase soy broth, with 5% sheep blood agar, Anaerobic condition, 37°C
Preincubation in aerobic condition in marine broth, with 5% sheep blood, Aerobic condition, 37°C
Preincubation in anaerobic condition in marine broth, with 5% sheep blood agar, Anaerobic condition, 37°C
Preincubation in aerobic condition in Brain Heart Infusion broth with 5% sheep blood, Aerobic condition, 37°C
Preincubation in anaerobic condition in Brain Heart Infusion broth with 5% sheep blood, Aerobic condition, 37°C

Supplementary Table 2. Bacterial species lost* at least 7 times by ethanol; * the list of all bacterial species lost at least once is available in Appendix 2.

**Species**	**Loss frequency**
Clostridium clostridioforme	-9
Bacteroides nordii	-8
Butyricimonas phoceensis	-8
Holdemania massiliensis	-8
Peptoniphilus grossensis	-8
Phascolarctobacterium faecium	-8
Alistipes indistinctus	-7
Bilophila wadsworthia	-7
Clostridium innocuum	-7
Olsenella uli	-7
Parabacteroides merdae	-7
Polynesia massiliensis	-7

Supplementary Table 3. Bacterial species added* at least 3 times by ethanol; *the list of all bacterial species gained at least once is available in Appendix 2.

**Species**	**Frequency of addition**
Massiliimalia timonensis	5
Propionimicrobium lymphophilum	4
Anaerotruncus massiliensis	3
Beduinibacterium massiliense	3
Clostridium saudimassiliensis	3
Massiliimalia massiliensis	3
Phocea massiliensis	3
Provencibacter massiliensis	3
Ruminiclostridium massiliense	3
Ruminococcus merdae	3
Ruthenibacterium lactatiformans	3

Supplementary Table 4. Information on fecal transplant donors whose samples stools were used in this study

Samples	Donor age	Donor sex	Body mass index (kg/m^2)	Nationality of fecal donors
Fresh stool 1	28	F	22.04	France
Fresh stool 2	32	M	22.22	Senegal
Fresh stool 3	32	M	22.45	Senegal
Fresh stool 4	26	F	17.98	Benin
Fresh stool 5	32	M	26.54	Cameroon
Fresh stool 6	30	M	23.59	France
Fresh stool 7	27	F	25.71	Algeria
Fresh stool 8	27	M	25.88	Algeria
Fecal infusion 1	28	F	22.04	France
Fecal infusion 2	26	F	17.98	Benin
Fecal infusion 3	75	F	29.30	France

Supplementary Table 5. Microbiological tests performed on stool and blood of the fecal transplant donor

**BACTERIOLOGY**	**Contraindication if mismatch with recipient**	**Possible transplant for HIV negative recipient**	**Qualification criteria**	**Non-qualification criteria**
Serology amoebiasis			Negative ☐	Positive ☐
Serology Trichinosis			Negative ☐	Positive ☐
Serology anguillulosis			Negative ☐	Positive ☐
Serology HBV			Negative ☐<br>Vaccine Profile ☐<br>Healed profile ☐	HbsAg Positive ☐<br>Hbc AcPositive isolated ☐
Viral load HBV			Negative ☐	Positive ☐
Serology HCV			Negative ☐	Positive ☐
Viral load HCV			Negative ☐	Positive ☐
Serology CMV	Positive ☐	Negative ☐		
Serology EBV	Positive ☐	Negative ☐		
Serology HTLV			Negative ☐	Positive ☐
Serology HIV			Negative ☐	Positive ☐
Viral load HIV			Negative ☐	Positive ☐
Serology Syphilis			Negative ☐	Positive ☐
Serology Toxoplasma gondii	Positive ☐	Negative ☐		
PCR stool Picornavirus (Rhino ou Enterovirus)			Negative ☐	Positive ☐
PCR stool HAV			Negative ☐	Positive ☐
PCR stool HEV			Negative ☐	Positive ☐
PCR Rotavirus			Negative ☐	Positive ☐
PCR Astrovirus			Negative ☐	Positive ☐
PCR Adenovirus (ADV)			Negative (<500) ☐	Positive (>500) ☐
PCR Calicivirus (Noro – Sapovirus)			Negative ☐	Positive ☐
Culture stool *Campylobacter*			Negative ☐	Positive ☐
Culture stool *Salmonella*			Negative ☐	Positive ☐
Culture stool *Shigella*			Negative ☐	Positive ☐
Culture stool *Yersinia*			Negative ☐	Positive ☐
Culture stool *Listeria monocytogenes*			Negative ☐	Positive ☐
Culture stool *Vibrio cholerae, V. parahaemolyticus*			Negative ☐	Positive ☐
Culture stool carbapenemase			Negative ☐	Positive ☐
Culture stool Extended spectrum beta-lactamase			Negative ☐	Positive ☐
Culture stool *Enterococcus* vancoR			Negative ☐	Positive ☐
Culture stool *Clostridium difficile*			Negative ☐	Positive ☐
PCR Shigatoxine 1 et 2			Negative ☐	Positive ☐
PCR stool *Entamoeba histolytica*			Negative ☐	Positive ☐
PCR stool *Strongyloides stercoralis*			Negative ☐	Positive ☐
PCR stool *Toxoplasma gondii*			Negative ☐	Positive ☐
PCR stool *Giardia intestinalis*			Negative ☐	Positive ☐
PCR stool *Cryptosporidium sp.*			Negative ☐	Positive ☐
PCR stool *Cyclospora sp.*			Negative ☐	Positive ☐
PCR stool *Isospora sp.*			Negative ☐	Positive ☐
PCR *Enterocytozoon bieneusi* (microsporidia)			Negative ☐	Positive ☐
PCR *Ancylostoma duodenale*			Negative ☐	Positive ☐
PCR *Ascaris lumbricoides*			Negative ☐	Positive ☐
PCR *Encephal.intesti*			Negative ☐	Positive ☐
PCR *Necator americanus*			Negative ☐	Positive ☐
PCR *Enterobius vermicularis*			Negative ☐	Positive ☐
PCR *Schistosoma mansoni*			Negative ☐	Positive ☐
PCR *Taenia saginata*			Negative ☐	Positive ☐
PCR *Taenia solium*			Negative ☐	Positive ☐
PCR *Trichuris trichiura*			Negative ☐	Positive ☐
PCR stool toxine B, binary , gene 027 C. difficile			Negative ☐	Positive ☐
Coprotheque (conservation 2 years)			☐	
Metagenomic 16S				

Appendices legends

Appendix 1. Classification of bacterial species according to oxygen tolerance, family, phylum and initial origin.

Sheet 1 : Total number of samples before ethanol disinfection

Sheet 2 : Total number of samples after ethanol disinfection

Sheet 3 : Fresh stools before ethanol disinfection

Sheet 4 : Infusions stools before ethanol disinfection

Sheet 5 : Fresh stools before ethanol disinfection

Sheet 6 : Infusions stools before ethanol disinfection

-= strict anaerobic ; += oxygen-tolerant ; H=Human ; NH=Non-Human ; H (gut)= Human-gut ; NS= Culturomics's new species isolated in previously study ; NS*= Culturomics's new species isolated after ethanol disinfection in this study.

Appendix 2. Microarray representing the overall impact of ethanol disinfection on each species and according to each sample. A gain corresponds to a species absent before disinfection but recovered after disinfection (+1), while a loss corresponds to a species present before disinfection but absent following disinfection (-1). Species recovered both before and after disinfection are indicated by a null value (0). FS= Fresh stool ; IF= Stool infusion; OH=ethanol ; na=not available

Appendix 3.

Sheet 1 : Classification of bacterial species found in previously bacteriotherapy trials by phylum, family and genera

Sheet 2 : Separate comparison of bacterial species shared between previous bacteriotherapy studies and our study

Sheet 3 : Grouped comparison of bacterial species shared between previous bacteriotherapy studies and our study

Sheet 4 : Grouped comparison of bacterial genera shared between previous bacteriotherapy studies and our study

Sheet 5 : Classification of bacterial species (unique to our strategy) following their oxygen tolerance, phylum and their initially origin.

Appendix 4.

Sheet 1 : Percentage of distribution by family of bacterial species isolated before disinfection with ethanol

Sheet 2 : Percentage of distribution by family of bacterial species isolated before disinfection with ethanol
